# Directions for Optimization of Photosynthetic Carbon Fixation: RuBisCO's Efficiency May Not Be So Constrained After All

**DOI:** 10.3389/fpls.2018.00183

**Published:** 2018-03-01

**Authors:** Peter L. Cummins, Babu Kannappan, Jill E. Gready

**Affiliations:** Gready Group, Department of Genome Science, John Curtin School of Medical Research, Australian National University, Canberra, ACT, Australia

**Keywords:** RuBisCO, carbon fixation, photosynthesis, enzyme kinetics and specificity, protein evolution, evolutionary constraints, enzyme-complex stability, gas-substrate binding

## Abstract

The ubiquitous enzyme Ribulose 1,5-bisphosphate carboxylase-oxygenase (RuBisCO) fixes atmospheric carbon dioxide within the Calvin-Benson cycle that is utilized by most photosynthetic organisms. Despite this central role, RuBisCO's efficiency surprisingly struggles, with both a very slow turnover rate to products and also impaired substrate specificity, features that have long been an enigma as it would be assumed that its efficiency was under strong evolutionary pressure. RuBisCO's substrate specificity is compromised as it catalyzes a side-fixation reaction with atmospheric oxygen; empirical kinetic results show a trend to tradeoff between relative specificity and low catalytic turnover rate. Although the dominant hypothesis has been that the active-site chemistry constrains the enzyme's evolution, a more recent study on RuBisCO stability and adaptability has implicated competing selection pressures. Elucidating these constraints is crucial for directing future research on improving photosynthesis, as the current literature casts doubt on the potential effectiveness of site-directed mutagenesis to improve RuBisCO's efficiency. Here we use regression analysis to quantify the relationships between kinetic parameters obtained from empirical data sets spanning a wide evolutionary range of RuBisCOs. Most significantly we found that the rate constant for dissociation of CO_2_ from the enzyme complex was much higher than previous estimates and comparable with the corresponding catalytic rate constant. Observed trends between relative specificity and turnover rate can be expressed as the product of negative and positive correlation factors. This provides an explanation in simple kinetic terms of both the natural variation of relative specificity as well as that obtained by reported site-directed mutagenesis results. We demonstrate that the kinetic behaviour shows a lesser rather than more constrained RuBisCO, consistent with growing empirical evidence of higher variability in relative specificity. In summary our analysis supports an explanation for the origin of the tradeoff between specificity and turnover as due to competition between protein stability and activity, rather than constraints between rate constants imposed by the underlying chemistry. Our analysis suggests that simultaneous improvement in both specificity and turnover rate of RuBisCO is possible.

## Introduction

Ribulose 1,5-bisphosphate carboxylase-oxygenase (RuBisCO) is the enzyme responsible for the fixation of carbon derived from atmospheric CO_2_ as part of the Calvin-Benson cycle that leads to production of the glucose essential for growth in most photosynthetic organisms. However, RuBisCO has a low turnover rate in higher plants (~3 s^−1^) and the efficiency of carbon fixation by the enzyme is compromised by a competing reaction with atmospheric O_2_ that leads to photorespiration at high cost to the organism in terms of both energy and loss of carbon. A recent analysis of *k*_*cat*_ and *K*_*M*_ values of several thousand enzymes (Bar-Even et al., 2011) has shown that RuBisCO's catalytic rate, *k*_*cat*_, and efficiency (*k*_*cat*_/*K*_*M*_) are not unusually low compared with values of the “average” enzyme (see their Figure 1), even though much lower than fast enzymes at the diffusion-controlled limit, for a variety of reasons including absence of strong evolutionary selection pressure and substrate properties, especially low molecular mass and hydrophobicity, limiting *K*_*M*_ optimization. A later analysis (Bar-Even et al., 2015) showed that enzyme-substate encounters for the “average” enzyme are not productive(“futile”), again for various reasons. The insights from these analyses are useful in placing RuBisCO's catalytic rate and efficiency in the context of all enzymes, especially the significant dissociation rate for CO_2_ we find in this work, but nonetheless puzzles remain as RuBisCO has been subject to very strong evolutionary pressure.

**Figure 1:**
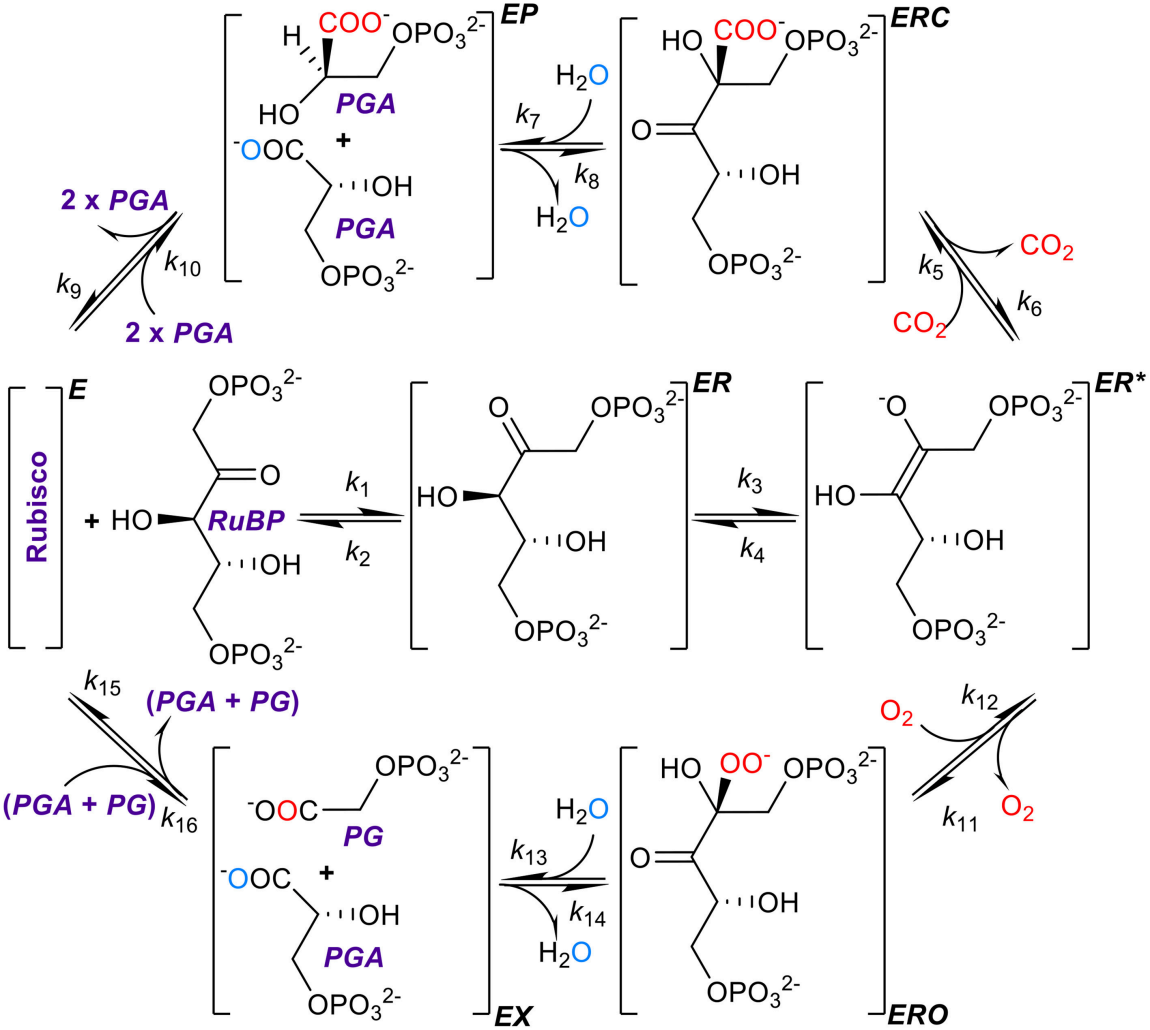
The kinetic mechanism of RuBisCO. RuBisCO must first be activated by carbamylation and binding of Mg^2+^ before it processes three substrates, ribulose bisphosphate (RuBP), and carbon dioxide or oxygen, the complete reactions taking place over several stages (Lorimer, 1981; Cleland et al., 1998; Andersson, 2008; Kannappan and Gready, 2008). RuBP binds first forming a complex (*ER*) with the activated form of the enzyme (*E*), followed by enolization of RuBP (ER^*^) which facilitates binding with the carbon dioxide or oxygen molecule to form the *ERC* or *ERO* enzyme-substrate complexes. After hydrolysis, the six-carbon compound formed by the addition of carbon dioxide to RuBP breaks at a C-C bond forming a product complex (*EP*) which dissociates into two three-carbon compounds, 3-phosphoglyceric acid (*PGA*), with the addition of two protons. Oxygenation proceeds through analogous steps except that the dissociation products are one *PGA* molecule and one of 2-phospho-glycolate (*PG*). Atoms originating from free CO_2_ and O_2_ are shown in red, and oxygen atom originating from the water molecule used for hydration is shown in aqua blue.

To mitigate this apparent torpidity of the enzyme, organisms have co-evolved other strategies for maintaining levels of photosynthesis. The observed large variations in RuBisCO kinetic parameters from photosynthetic organisms in different kingdoms down to different species (Jordan and Ogren, 1981, 1983) is a consequence of co-evolution with resource allocation into other strategies that lead to enhanced photosynthesis (largely by way of more efficient CO_2_ and nitrogen utilization) and suppressed photorespiration (Badger and Andrews, 1987; Badger et al., 1998).

Cyanobacterial RuBisCOs are characterized by lower values of activity with CO_2_ relative to that of O_2_ (the relative specificity, *S*_*C*/*O*_) and higher catalytic turnover rates ($k_{cat}^{C}$). These organisms utilize a carbon-concentrating mechanism (CCM) which compensates for the lower *S*_*C*/*O*_ and limits photorespiration by increasing the CO_2_/O_2_ ratio at the site of fixation, while taking advantage of the higher $k_{cat}^{C}$ by reducing RuBisCO concentration and hence the requirement for nitrogen. Some non-green algae with higher *S*_*C*/*O*_ do not express a CCM but instead the lower $k_{cat}^{C}$ is mitigated by increasing RuBisCO and, hence, higher investment of nitrogen in RuBisCO protein. In higher plants, the kinetic balances and photosynthetic pathways lie somewhere in the middle of these two extremes. In C_3_ plants *S*_*C*/*O*_ is generally greater and $k_{cat}^{C}\;$less than in C_4_ plants expressing CCMs (Yeoh et al., 1980; Seemann et al., 1984; Ghannoum et al., 2005), while others are characterized as C_3_-C_4_ intermediate or C_4_-like (Kubien et al., 2008).

Understanding the nature of constraints imposed on RuBisCO's intrinsic efficiency is important for directing future research on photosynthesis. Study of RuBisCO activity has become a focus for improving photosynthesis (Bainbridge et al., 1995; Peterhansel et al., 2008; Gready and Kannappan, 2009; Whitney et al., 2011; Parry et al., 2013; Carmo-Silva et al., 2015) with a major aim of improving crop yields. However, some doubt has been cast on whether it can be significantly improved via mutation because of a hypothesis of “underlying constraints” in the chemistry of the reaction (Tcherkez et al., 2006; Savir et al., 2010; Tcherkez, 2013).

In the present study, we argue that this conclusion may have resulted from unsupported assumptions of the kinetic models and limited data sets used in the analyses. Resolving the precise nature of the constraints imposed on RuBisCO kinetics is clearly pivotal to providing direction of future research into improving photosynthesis. The rate constants (Figure 1) determine, and therefore ultimately limit, the physical binding of substrates, the breaking and formation of chemical bonds, and finally the release of products (Lorimer, 1981; Cleland et al., 1998; Andersson, 2008; Kannappan and Gready, 2008).

Although methods for computing individual rate constants from kinetic data have not been widely implemented for RuBisCO (McNevin et al., 2006), the more commonly measured kinetic parameters ($k_{cat}^{C}$, $k_{cat}^{O}$, *K*_*C*_, *K*_*O*_, and $S_{C/O}=\frac{k_{cat}^{C}K_{O}}{k_{cat}^{O}K_{C}}$), *in vitro*, are generally functions of these. Here we derive the equations for the kinetic mechanism (Figure 1) and estimate the mean (or expected) values for rate constants using regression analysis. Utilizing the compilation in Table 1, which includes the data used by Savir et al. in their analysis (Savir et al., 2010), we performed our own linear regression analysis on a wider range of data sets. This analysis was extended to other plant data (Galmés et al., 2014; Prins et al., 2016) to assist in validating the results. We found that the rate constants for dissociation of the CO_2_ and O_2_ substrates (*k*_6_ and *k*_12_ in Figure 1) are much larger relative to the corresponding catalytic rate than previously assumed and consequently have a significant effect on the kinetics. We also suggest the constraints on RuBisCO may be better explained by competing selection pressures, rather than by positive selection within hypothetical constraints (Tcherkez et al., 2006; Tcherkez, 2013) imposed by the chemical mechanism.

**Table 1:** RuBisCO kinetic parameters.

**Species**	**Ref**.	**$k_{cat}^{C}$ (s^−1^)**	**$k_{cat}^{O}$ (s^−1^)**	***S*_*C*/*O*_(mol/mol)**	***K*_*O*_ (μM)**	***K*_*C*_ (μM)**
Higher plant C_3_ (*Triticum aestivum*)	a	2.5	1.45	90	730	14
Higher plant C_4_-like (*Flareria brownie*)	b	2.58	0.91	83.8	378	12.8
Higher plant C_3_-C_4_ (*Flaveria sonorensis*)	b	2.69	2.46	84.3	785	10.2
Higher plant C_3_-C_4_ (*Flaveria ramosissima*)	b	2.77	2.09	79.8	722	12.0
Higher plant C_3_-C_4_ (*Flaveria angustifolia*)	b	2.86		83.2		13.1
Higher plant C_3_ (*Chenopodium alba*)	a	2.91	1.37	78.7	415	11.2
Higher plant C_3_ (*Flaveria pringlei*)	a	3.1	2.14	80.8	666	12.0
Higher plant C_4_ (*Paspalum dilatatum)*	c	3.11	0.74	88	415	19.9
Higher plant C_3_ (*Flaveria cronquistii*)	b	3.13	2.34	81	653	10.8
Higher plant C_3_-C_4_ (*Flaveria floridana*)	b	3.19	1.96	84.5	686	13.2
Higher plant C_3_ (*Spinacia oleracea*)	b	3.20	1.90	79.8	574	12.1
Higher plant C_3_-C_4_ (*Flaveria chloraefolia*)	b	3.35	2.45	81.6	740	12.4
Higher plant C_3_ (*Nicotiana tabacum*)	a	3.4	1.11	82	295	10.7
Higher plant C_4_ (*Cynodon dactylon)*	c	3.41	0.73	89	402	21
Higher plant C_3_-C_4_ (*Flaveria linearis*)	b	3.43	1.46	78.1	415	12.5
Higher plant C_4_-like (*Flaveria palmeri*)	b	3.54	0.60	83.8	193	13.5
Higher plant C_4_ (*Flaveria kochiana*)	b	3.68	0.32	77	150	22.7
Higher plant C_3_ (*Spinacia oleracea*)	a	3.7	1.59	80	480	14
Higher plant C_4_-like (*Flaveria vaginata*)	b	3.78	1.98	78.7	880	21.4
Higher plant C_4_ (*Zoysia japonica)*	c	3.78	0.98	84.1	403	18.5
Higher plant C_4_ (*Amaranthus hybridus*)	a	3.8	1.85	82	640	16
Higher plant C_4_ (*Flaveria australasica*)	a	3.84	0.70	77.2	309	22.0
Higher plant C_4_ (*Zea mays*)	d	4.05	0.32	74.9	157	26.2
Higher plant C_4_ (*Amaranthus edulis*)	a	4.14	0.85	77.5	289	18.2
Higher plant C_4_ (*Flaveria bidentis*)	b	4.16	1.74	75.5	639	20.2
Higher plant C_4_ (*Zea mays*)	a	4.4	1.34	78	810	34
Higher plant C_4_ (*Flaveria trinervia*)	b	4.42	2.15	77	671	17.9
Higher plant C_4_ (*Sorghum bicolor*)	a	5.4		70		30
Higher plant C_4_ (*Zea mays*)	d	5.5	1.31	88	397	19
Higher plant C_4_ (*Potulaca oleraca*)	a	5.9		78		13.6
Green algae (*Chlamydomonas reinhardtii*)	a	5.8	1.57	61	480	29
Cyanobacteria (*Synechococcus* 6301)	a	11.6	0.77	43	972	340
Cyanobacteria (*Synechococcus* 7002)	a	13.4	1.36	52	1300	246
Nongreen algae (*Cylindrotheca* sp. N1)	e	0.78		106	1292	31
Nongreen algae (*Olisthodiscus luteus*	e	0.83		101	692	59
Nongreen algae (*Galdieria sulfuraria*)	a	1.2	0.82	166	374	3.3
Nongreen algae (*Cyanidium caldarium*	e	1.3		224		6.7
Nongreen algae *Porphyridium cruentum*	e	1.6		129	1574	22
Nongreen algae *Cyanidium partita*	e	1.6		238		6.6
Nongreen algae *Cylindrotheca fusiformis*	e	1.95		110	568	36
Nongreen algae (*Griffithsia monilis*)	a	2.6		167		9.3
Nongreen algae (*Phaeodactylum tricornutum*)	a	3.4	0.50	113	467	28
Diatom (*Bellerochea cf. horologicalis*)	d	2.1			764	50
Diatom (*Thalassiosira oceania*)	d	2.4	0.44	80	954	65
Diatom (*Chaetoceros muelleri*)	d	2.4	0.46	96	425	23
Diatom (*Chaetoceros calcitrans*)	d	2.6	0.75	57	413	25
Diatom (*Phaeodactylum tricornutum*)	d	3.2	0.49	108	592	36
Diatom (*Skeletonema marinoi*)	d	3.2			883	68
Diatom (*Thalassiosira weissflogii*)	d	3.2	1.27	79	2032	65
Diatom (*Phaeodactylum tricornutum*)	d	3.3	0.46	116	664	41
Diatom (*Chaetoceros calcitrans*)	d	3.4	0.72	75	490	31
Diatom (*Fragilariopsis cylindrus*)	d	3.5	0.47	77	667	64
Diatom (*Cylindrotheca fusiformis*)	d	3.7		79		
Bacteria (*Chromatium vinosum*)	a	6.7	1.28	41	290	37
Bacteria (*Rhodospirillum rubrum*)	a	7.3	3.01	12.3	406	80

*Data compiled by Savir et al. (2010) are highlighted in green*.

*a Savir et al. (2010); b Kubien et al. (2008); c Carmo-Silva et al. (2010); d Young et al. (2016); e Badger et al. (1998)*.

Our results and conclusions are indicative of a less constrained RuBisCO and are consistent with observed variations in the kinetics of a wider range of wild type and mutant RuBisCO that are now available, although such kinetic data is regrettably still sparse.

## Methods

We consider the rate constants *k*_*i*_ for the kinetic mechanism (Figure 1) to be a set of general random variables (Koralov and Sinai, 2007). The expected value, *E*(*k*_*i*_) ≡ 〈*k*_*i*_〉, is the mean value of *k*_*i*_, i.e., averaged over a number of sequences. In principle these averages can be extracted using both linear and non-linear regression methods to establish functional relationships between the RuBisCO kinetic parameters. As *K*_*C*_ and *K*_*O*_ depend explicitly on $k_{cat}^{C}$ and $k_{cat}^{O}$, respectively, we restrict the independent variables (predictors) to $k_{cat}^{C}$ and $k_{cat}^{O}$. The dependent (response) variables whose expected values, conditional on $k_{cat}^{C}$ or $k_{cat}^{O}$, are determined by regression are then *K*_*C*_, *K*_*O*_ and *S*_*C*/*O*_, e.g., $E\left(K_{C}|k_{cat}^{C}\;\right)\equiv \langle K_{C}\rangle$.

The Generalized Extreme Studentized Deviate (ESD) test (Rosner, 1983) was used with *P*-value of 0.05 to eliminate multiple outliers in the data prior to regression analysis. The regression parameters were then used to estimate the expected values of various terms in the kinetic equations. We can illustrate the procedure by considering a more simplistic single-intermediate kinetic mechanism where the Michaelis constant is given by $K_{M}=\frac{\left(k_{cat}+k_{off}\right)}{k_{on}}$ (e.g, Roberts, 1977; Farquhar, 1979). Enzyme assays typically provide *K*_*M*_ and *k*_*cat*_ but insufficient data to determine *k*_*on*_ and *k*_*off*_ which are, respectively, the rate constants for the binding and dissociation of substrate (e.g., CO_2_ or O_2_). However, if we consider that the rate constants *k*_*cat*_, *k*_*on*_ and *k*_*off*_ randomly fluctuate over a number of sequences, a linear correlation, 〈*K*_*M*_〉, may be obtained between *K*_*M*_ and *k*_*cat*_ from which the gradient and intercept give the expected values $\langle \frac{1}{k_{on}}\rangle$ and $\langle \frac{k_{off}}{k_{on}}\rangle$, respectively, and using the approximation 〈*xy*〉 ≈ 〈*x*〉〈*y*〉 for a finite number of random variables *x* and *y*, we can hence determine the expected values of the rate constants 〈*k*_*on*_〉 and 〈*k*_*off*_〉. Although *K*_*M*_ is linearly dependent on *k*_*cat*_, we should not necessarily expect to observe any correlation, as high variances may be associated with the other two terms, *k*_*on*_ and *k*_*off*_. Where a linear correlation exists, we may infer that the rate constants *k*_*on*_ and *k*_*off*_ are fairly constant (low variance), while a non-linear correlation would be consistent with an additional correlation between *k*_*cat*_ and at least one of these other two terms. Statistical (regression) methods are here used to show how these different scenarios are represented in the available kinetic data.

## Results

### Kinetic equations

In deriving the following kinetic equations for this mechanism (Figure 1) we assumed only that both *k*_10_ and *k*_16_ are very much smaller than any of the remaining rate constants (effectively, *k*_10_ = *k*_16_ = 0). We emphasize that no such approximations (*k*_*i*_ = 0) were made anywhere else in the derivation. The Michaelis constants (*K*_*M*_) for carboxylation and oxygenation are then given, respectively, by equations of the form (Equations A23, A24; see Appendix in Supplementary Materials for details of derivations)

(1) $$\begin{array}{l} K_{C}=\frac{\left(k_{cat}^{C}+\gamma_{C}k_{6}\right)}{K_{R}k_{5}} \end{array}$$

(2) $$\begin{array}{l} K_{O}=\frac{\left(k_{cat}^{O}+\gamma_{O}k_{12}\right)}{K_{R}k_{11}} \end{array}$$

The general equation for the specificity of carboxylation relative to that of oxygenation (relative specificity) is then (Equation A25).

(3) $$\begin{array}{l} S_{C/O}=\frac{S_{C}}{S_{O}}=\frac{k_{cat}^{C}K_{O}}{K_{C}k_{cat}^{O}}=\frac{k_{5}k_{cat}^{C}\left(k_{cat}^{O}+\gamma_{O}k_{12}\right)}{k_{11}k_{cat}^{O}\left(k_{cat}^{C}+\gamma_{C}k_{6}\right)} \end{array}$$

In Equation (3), the relative specificity (*S*_*C*/*O*_) is formally a function of 10 rate constants (*k*_5_..*k*_9_, *k*_11_..*k*_15_), five for each of the carboxylation and oxygenation reactions. $K_{R}=\frac{k_{3}}{\left(k_{3}+k_{4}\right)}$ is a function only of rate constants for the enolization step (Equation A22), i.e., independent of carboxylation or oxygenation, and 0 < γ < 1. Both $k_{cat}^{C}$ and γ_*C*_ are formally functions of *k*_3_, *k*_7_, *k*_8_ and *k*_9_ (Equation A26). It is evident (Equation A26) that if *k*_7_ is the slow step that determines the maximum catalytic rate ($k_{cat}^{C}=k_{7}$), then γ_*C*_ = 1. Similarly (Equation A27), if $k_{cat}^{O}=k_{13}$, then γ_*O*_ = 1. However, we need not make these types of assumptions here, and simply regard γ_*C*_*k*_6_ and γ_*O*_*k*_12_ as effective dissociation rate constants.

### Michaelis constants

**Figure 2:**
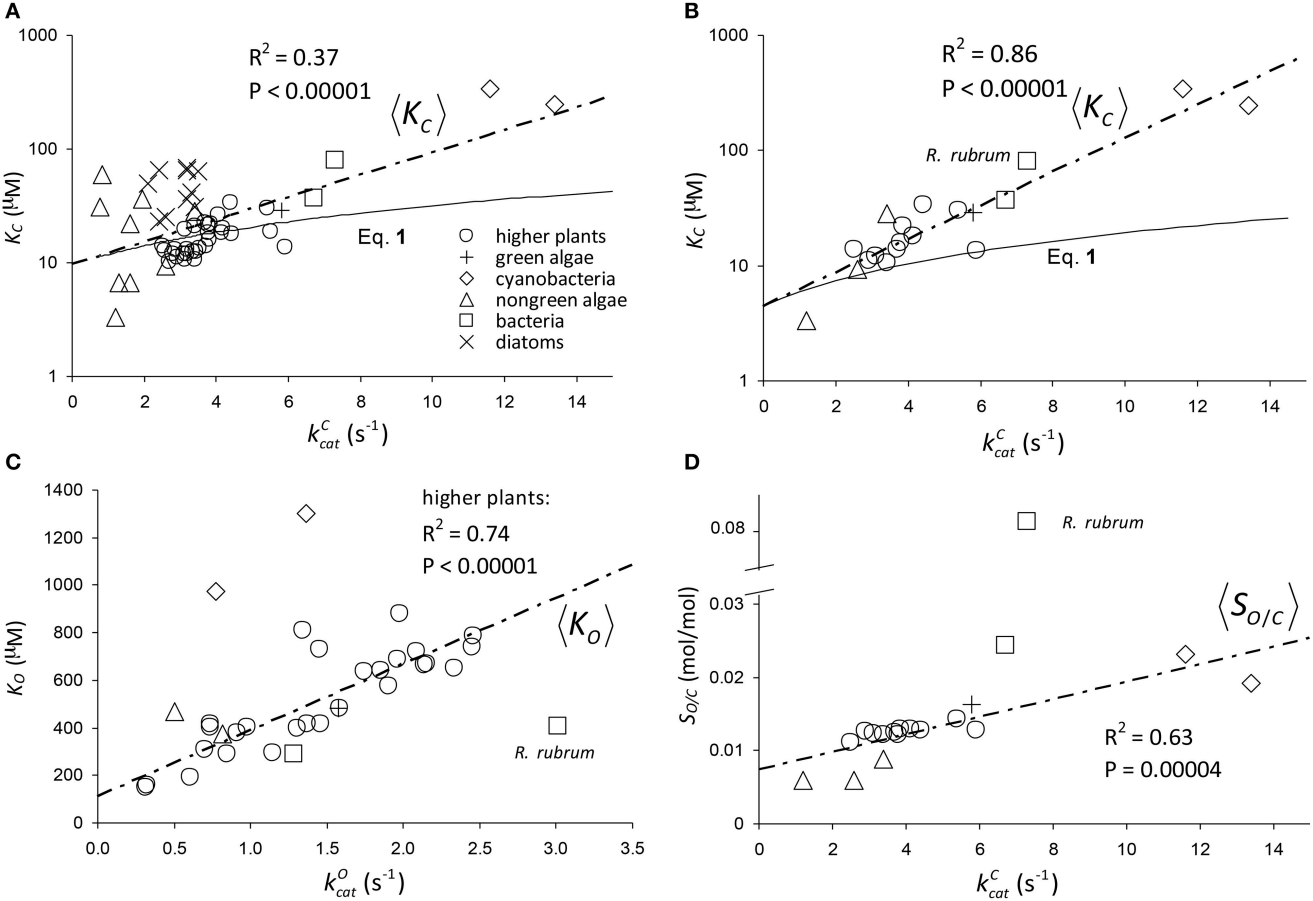
Regression of: **(A)** *K*_*C*_ on $k_{cat}^{C}$ using all data (Table 1) in the regression. The parameters of the exponential, $a_{1}e^{b_{1}k_{cat}^{C}}$, are *a*_1_ = 9.7 μM and *b*_1_ = 0.23*s*. **(B)** *K*_*C*_ on $k_{cat}^{C}$ using only the data compiled by Savir et al. (2010) in the regression. The parameters of the exponential are *a*_1_ = 4.5 μ*M* and *b*_1_ = 0.34*s*. The Form II RuBisCO, *R. rubrum*, is not a significant outlier. **(C)** *K*_*O*_ on $k_{cat}^{O}$ using all higher plant data (Table 1) only. The gradient and intercept of the regression line are 278 μM.s and 114 μM, respectively. Form II RuBisCO, *R. rubrum*, and the cyanobacteria are the significant outliers by the ESD test with *P* = 0.05 (Rosner, 1983). **(D)** Reciprocal relative specificity ($S_{O/C}=\frac{1}{S_{C/O}\;}$) on $k_{cat}^{C}$ using the data compiled by Savir et al. (2010). The Form II RuBisCO, *R. rubrum* is the only significant outlier by the ESD test (*P* = 0.05), due mainly to its relatively higher value for $S_{O}=\frac{k_{cat}^{O}}{K_{O}}$ (Figure 2C), and was not included in the regression. The gradient and intercept are 1.2 × 10^−3^*s* and 7.4 × 10^−3^*mol/mol*, respectively. Note that in **(A,B)** *K*_*C*_ is graphed in logarithmic scale and Equation (1) has been graphed using the parameters at $k_{cat}^{C}=0$ as derived from the regression analysis (see text).

**Figure 3:**
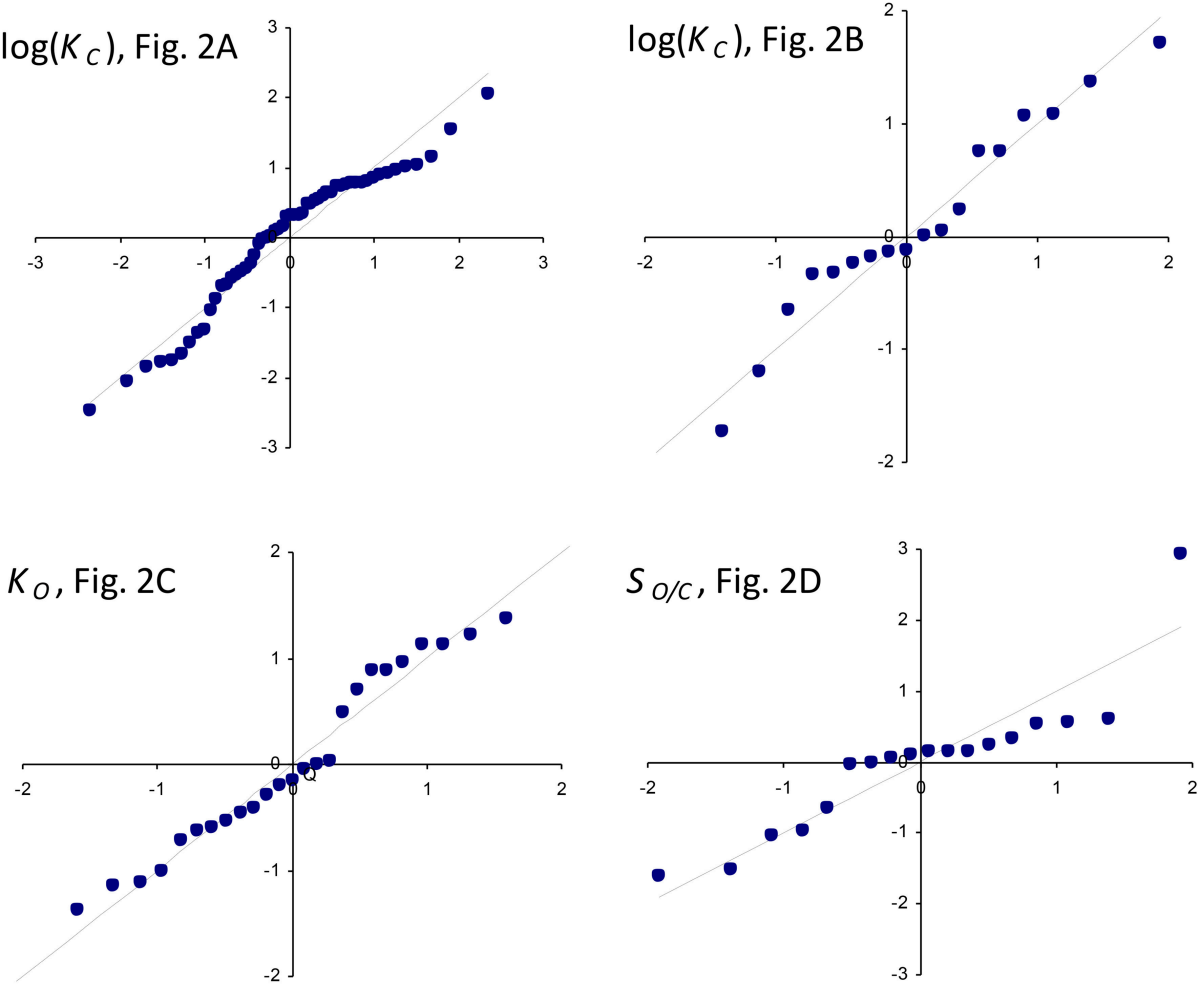
Normal Q-Q standardized plots of residuals for log(*K*_*C*_) (Figures 2A,B), *K*_*O*_ (Figure 2C), and the reciprocal relative specificity, $S_{O/C}=\frac{1}{S_{C/O}\;}$ (Figure 2D).

**Figure 4:**
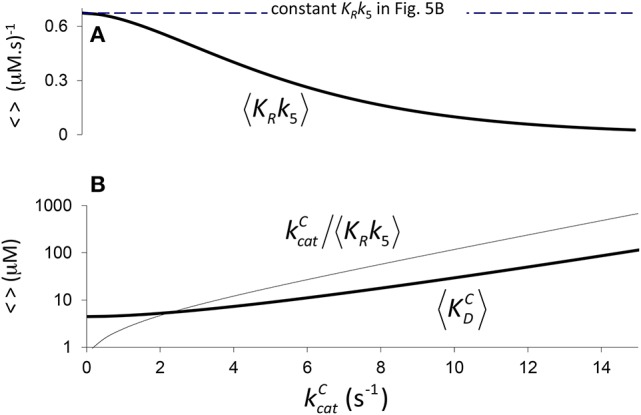
Components of *K*_*C*_ if correlation is due to CO_2_ binding. **(A)** 〈*K*_*R*_*k*_5_〉 Equation (4) calculated assuming a constant value of $\langle \gamma_{C}k_{6}\rangle =3s^{-1}$ (Figure 2B, *a*_1_ = 4.5 μM, *b*_1_ = 0.34 *s*), and **(B)** the corresponding components of 〈*K*_*C*_〉, i.e. $\frac{k_{cat}^{C}}{\langle K_{R}k_{5}\rangle }$ and $\langle K_{D}^{C}\rangle$ Equation (5), derived from Equation (1) ($\langle K_{C}\rangle =\frac{k_{cat}^{C}}{\langle K_{R}k_{5}\rangle }+\langle K_{D}^{C}\rangle$). **(B)** only is graphed in logarithmic scale.

The results of linear regression analysis performed on a number of data sets are summarized in Table 2. The green algae, bacteria and cyanobacteria data in Table 1 and other plant species (Galmés et al., 2014) could not be considered individually for analysis due to the small numbers of observations (*N* < 3). The log-scale plots (Figures 2A,B) of *K*_*C*_ over the full range of $k_{cat}^{C}$ values in Table 1 suggest a linear correlation and hence regression analysis of ln(*K*_*C*_) on $k_{cat}^{C}$ (“All data” sets in Table 2). *P* < 0.05 for both coefficients were obtained only for carboxylation using the “All data” sets (Figures 2A,B), carboxylation using a subset of the C_3_ plants (Galmés et al., 2014), oxygenation using Triticeae data (Prins et al., 2016) and oxygenation using only the higher plant data (Figure 2C). The residuals were found to be near-normally distributed (Figure 3). Reliable expected values for effective CO_2_ and O_2_ dissociation rate constants can be derived from the coefficients in regressions (Table 2) that yield *P* < 0.05 for both coefficients (i.e., both the gradient and intercept). The results are given in Table 3. For the regression of ln(*K*_*C*_) on $k_{cat}^{C}$, equating the first terms ($a_{1}+a_{1}b_{1}k_{cat}^{C}$) in the expansion of the exponential form ($a_{1}e^{b_{1}k_{cat}^{C}}$) with Equation (1) we find that the value of *K*_*C*_ at $k_{cat}^{C}=0$ is given by $\langle \frac{\gamma_{C}k_{6}}{K_{R}k_{5}}\rangle =a_{1}$. From the regression analysis carried out using the full data set in Table 1 (Figure 2A) and the subset utilized by Savir et al. (2010) (Figure 2B), we obtain values of *a*_1_ = 9.7 μ*M* and *a*_1_ = 4.5 μ*M*, respectively. From the expansion of the exponential we also find that $\langle \frac{1}{K_{R}k_{5}}\rangle \approx a_{1}b_{1}$ at $k_{cat}^{C}=0$, where the two estimates are *a*_1_*b*_1_ = 2.2 μ*M.s* and *a*_1_*b*_1_ = 1.5 μ*M.s*, respectively. In Figures 2A,B, Equation (1), which will obviously deviate from the trend line as $k_{cat}^{C}$ increases, has been graphed using these values. Combining these results obtained for $\langle \frac{\gamma_{C}k_{6}}{K_{R}k_{5}}\rangle$ and $\langle \frac{1}{K_{R}k_{5}}\rangle$ we estimate (at $k_{cat}^{C}=0$) expected effective rate constants for CO_2_ dissociation (〈γ_*C*_*k*_6_〉) of 4.3*s*^−1^ and 3.0*s*^−1^, respectively. Assuming the scheme (Figure 1) correctly describes the kinetic mechanism, the deviation from linear behavior suggests there exists at least one type of correlation between rate constants. From Equation (1), the expected value of *K*_*R*_*k*_5_ conditional on $k_{cat}^{C}$ in terms of regression parameters *a*_1_ and *b*_1_ is then given by (Figure 4A).

(4) $$\begin{array}{l} \langle K_{R}k_{5}\rangle =\frac{\left(k_{cat}^{C}+\langle \gamma_{C}k_{6}\rangle \right)}{a_{1}e^{b_{1}k_{cat}^{C}}}. \end{array}$$

**Table 2:** Linear regressions of *K*_*M*_ or ln(*K*_*M*_) on *k*_*cat*_ for various data sets of sample size *N*: Coefficients of *y*-intercept, *K*_*M*_ or ln(*K*_*M*_), and *x*-variable (gradient), *k*_*cat*_, with standard errors (SE), *P*-values and 95% (*P* = 0.05) confidence intervals.

**Regression**	**Coefficients**		**SE**	***P*-value**	**Lower 95%**	**Upper 95%**
Other than C_3_ Plants^a^	*K*_*C*_	15.3	3.4	0.001	7.8	22.7
(*N* = 14, *P-value* = 0.39)	$k_{cat}^{C}$	−0.9	1.0	0.39	−3.0	1.3
C_3_ Plants^a^	*K*_*C*_	4.5	1.4	0.007	1.5	7.5
(*N* = 14, *P-value* = 0.008)	$k_{cat}^{C}$	1.4	0.4	0.008	0.4	2.4
C_3_ Plants^a^^,^^c^	*K*_*C*_	5.2	2.5	0.05	−0.04	10.4
(*N* = 21, *P-value* = 0.072)	$k_{cat}^{C}$	1.6	0.8	0.07	−0.2	3.3
Higher Plants^b^	*K*_*C*_	3.2	8.9	0.73	−17.0	23.5
(*N* = 11, *P-value* = 0.13)	$k_{cat}^{C}$	3.7	2.2	0.13	−1.3	8.7
Higher Plants^c^	*K*_*C*_	2.9	4.2	0.51	−5.8	11.5
(*N* = 30, *P-value* = 0.002)	$k_{cat}^{C}$	3.8	1.1	0.002	1.5	6.1
Non-green algae^c^	*K*_*C*_	28.6	15.0	0.10	−6.8	64.0
(*N* = 9, *P-value* = 0.66)	$k_{cat}^{C}$	−3.7	8.0	0.66	−22.5	15.2
Diatoms^c^	*K*_*C*_	26.8	36.6	0.49	−57.7	111
(*N* = 10, *P-value* = 0.60)	$k_{cat}^{C}$	6.8	12.3	0.60	−21.6	35.3
Triticeae^d^	*K*_*C*_	9.8	3.2	0.03	1.6	17.9
(*N* = 7, *P-value* = 0.15)	$k_{cat}^{C}$	1.8	1.1	0.15	−0.9	4.6
Triticeae^d^	*K*_*O*_	315	35.8	0.0003	223	408
(*N* = 7, *P-value* = 0.023)	$k_{cat}^{O}$	138	42.6	0.02	28.5	247
Higher Plants^c^ (Figure 2C)	*K*_*O*_	115	52.1	0.04	7.5	222
(*N* = 27, *P-value* < 10^−5^)	$k_{cat}^{O}$	278	33.1	<10^−5^	210	346
All Data^c^ (Figure 2A)	ln(*K*_*C*_)	2.3	0.2	<10^−5^	1.9	2.6
(*N* = 54, *P-value* < 10^−5^)	$k_{cat}^{C}$	0.23	0.04	<10^−5^	0.15	0.31
All Data^b^ (Figure 2B)	ln(*K*_*C*_)	1.5	0.2	<10^−5^	1.1	1.9
(*N* = 19, *P-value* < 10^−5^)	$k_{cat}^{C}$	0.34	0.03	<10^−5^	0.27	0.40

a Data from Table 1 in Galmés et al. (2014)

b Table 1 Savir et al. (2010)

c Table 1

d 25°C data from Table 2 in Prins et al. (2016)

**Table 3:** Expected values of dissociation rate constants (s^−1^) for carboxylation (γ_*C*_*K*_6_) and oxygenation (γ_*C*_*K*_12_) with standard errors and corresponding 95% confidence intervals calculated from coefficients (gradient and intercept) with *P* < 0.05 in Table 2.

**Rate constant**	**γ_C_K_6_^a^**	**γ_C_K_6_^b^**	**γ_C_K_6_^c^**	**γ_C_K_12_^d^**	**γ_C_K_12_^e^**
Expected value	4.4	3.0	3.2	0.4	2.3
Standard Error	±0.8	±0.3	±1.4	±0.2	±0.8
95% Confidence Interval	±1.6	±0.6	±3.0	±0.4	±1.9

a Table 1

b Savir et al. (2010) (Table 1)

c C_3_ plant data from Table 1 in Galmés et al. (2014)

d higher plants (Table 1)

e *25°C Triticeae data from Table 2 in Prins et al. (2016)*.

Therefore, we may also use Equation (4) to define the expected effective dissociation constant conditional on $k_{cat}^{C}$ as (Figure 4B).

(5) $$\begin{array}{l} \langle K_{D}^{C}\rangle =\frac{\langle \gamma_{C}k_{6}\rangle }{\langle K_{R}k_{5}\rangle }. \end{array}$$

**Figure 5:**
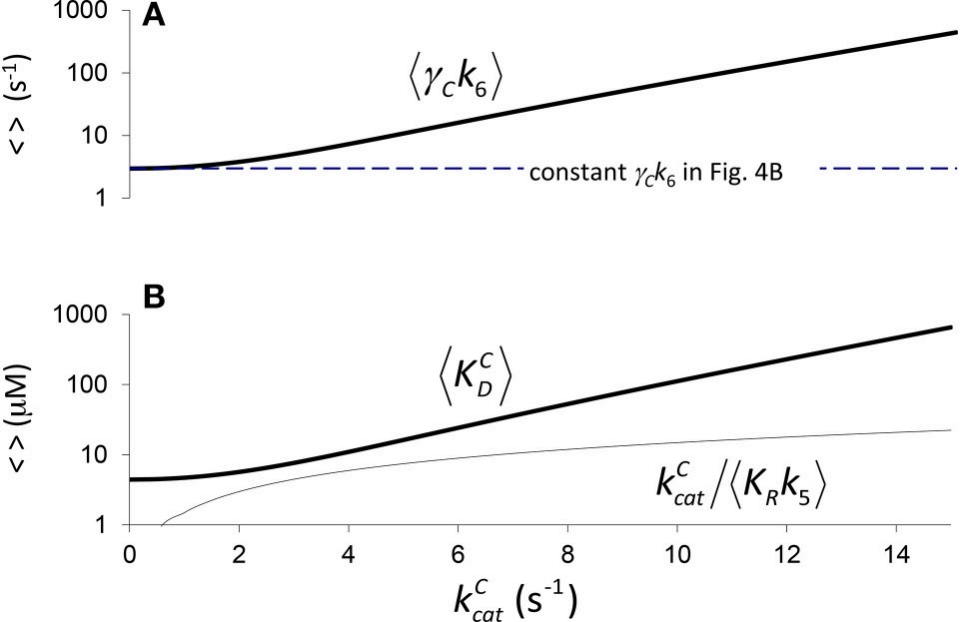
Components of *K*_*C*_ if correlation is due to CO_2_ dissociation. **(A)** $\langle \gamma_{C}k_{6}\rangle =\langle K_{R}k_{5}\rangle a_{1}e^{b_{1}k_{cat}^{C}}-k_{cat}^{C}$, (from rearranging Equation 4) calculated assuming a constant value of $\langle K_{R}k_{5}\rangle =0.7\;{\left(\mu \text{M}.\text{s}\right)}^{-1}$ (Figure 2B, *a*_1_ = 4.5 μM, *b*_1_ = 0.34 *s*), and **(B)** the corresponding components of 〈*K*_*C*_〉, i.e., $\frac{k_{cat}^{C}}{\langle K_{R}k_{5}\rangle }$ and $\langle K_{D}^{C}\rangle$. Both **(A,B)** are graphed in logarithmic scale.

In Figure 4 it is assumed (Tcherkez et al., 2006) that the exponential increase in 〈*K*_*C*_〉 conditional on $k_{cat}^{C}$ arises from 〈*K*_*R*_*k*_5_〉 (one correlation effect, i.e., due to CO_2_ binding) while 〈γ_*C*_*k*_6_〉 is a constant in Equation (4). Alternatively, in Figure 5 we have assumed that variation arises from 〈γ_*C*_*k*_6_〉 (another correlation effect i.e., due to CO_2_ dissociation) while 〈*K*_*R*_*k*_5_〉 is now the constant. Here the respective constants are the values of 〈γ_*C*_*k*_6_〉 and 〈*K*_*R*_*k*_5_〉 at $k_{cat}^{C}=0$ as determined from the regression (Figure 2B). There is, of course, also the possibility that variability in both 〈*K*_*R*_*k*_5_〉 and 〈γ_*C*_*k*_6_〉 contribute to the non-linear behavior of 〈*K*_*C*_〉, i.e., both 〈*K*_*R*_*k*_5_〉 and 〈γ_*C*_*k*_6_〉 are conditional on $k_{cat}^{C}$. In general, therefore, we could ascribe any functional dependence for either 〈*K*_*R*_*k*_5_〉 or 〈γ_*C*_*k*_6_〉 to this non-linear behavior.

For the regression of *K*_*O*_ on $k_{cat}^{O}$ (Figure 2C), we have included only the data for all higher plants (Table 1). Unlike the above regressions of *K*_*C*_ on $k_{cat}^{C}$ there are no indications of any deviations from non-linear behavior. The graph of *K*_*O*_ on $k_{cat}^{O}$ for the higher plants in particular clearly conforms to a linear function, and the residuals of regressed *K*_*O*_ data are near normally distributed (Figure 3). From the intercept we find the expected value of the dissociation constant

(6) $$\begin{array}{l} \langle K_{D}^{O}\rangle =\frac{\langle \gamma_{O}k_{12}\rangle }{\langle K_{R}k_{11}\rangle }\approx 110\;\mu \text{M} \end{array}$$

and from the gradient we obtain the constant

(7) $$\begin{array}{l} \langle \frac{1}{K_{R}k_{11}}\rangle \approx 280\;\mu \text{M}.\text{s}. \end{array}$$

From Equations (6, 7) we estimate the expected value of the effective O_2_ dissociation rate constant, $\langle \gamma_{O}k_{12}\rangle \approx 0.3\;{\text{s}}^{-1}$. Finally, from the above determinations of $\langle \frac{1}{K_{R}k_{5}}\rangle$ (from Figure 2B) and $\langle \frac{1}{K_{R}k_{11}}\rangle$ we can estimate the expected CO_2_ to O_2_ ratio of the rate constants for binding at $k_{cat}^{C}=0$ as $\langle \frac{k_{5}}{k_{11}}\rangle \approx 190$.

### Relative specificity

**Figure 6:**
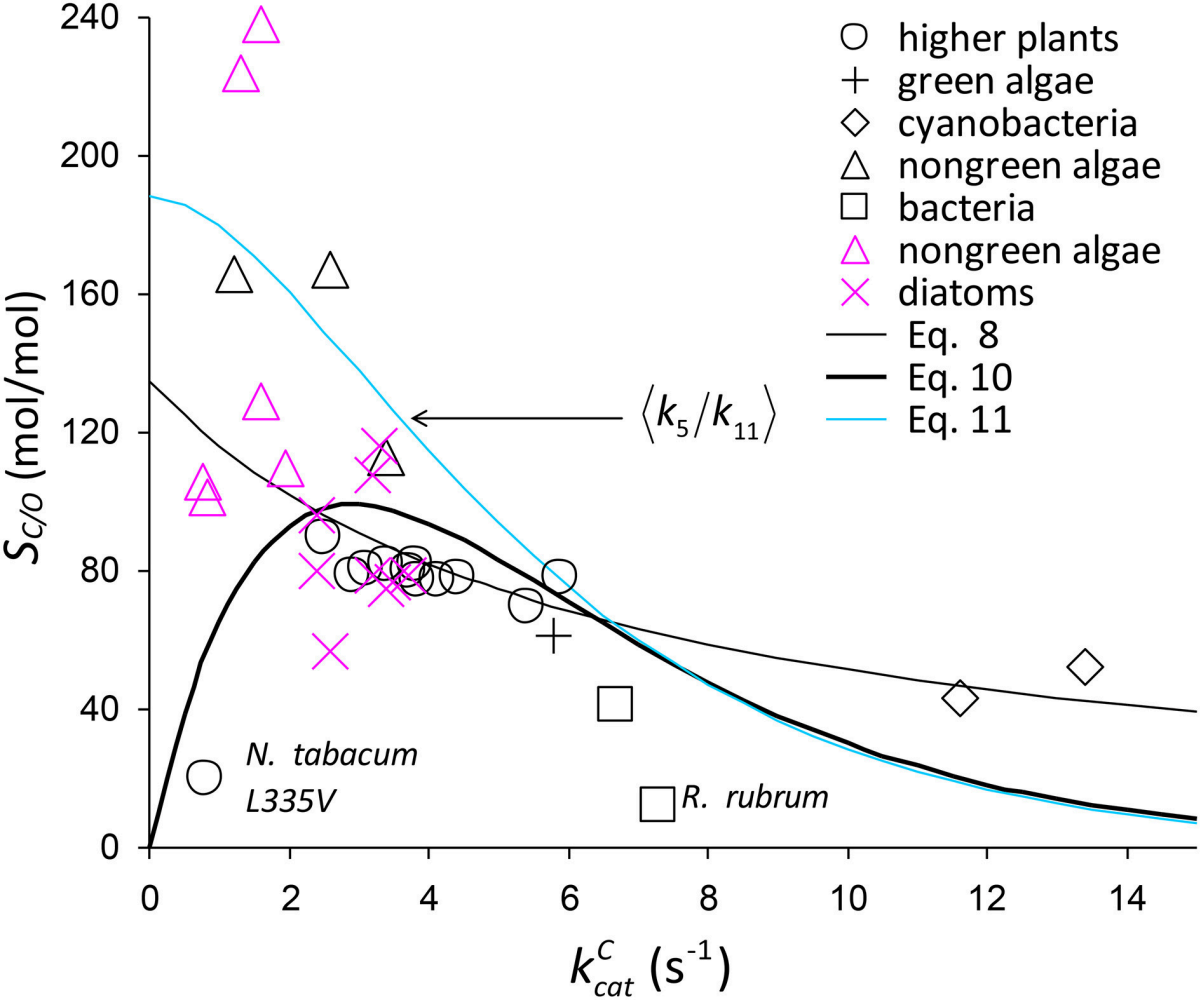
Selection of *S*_*C*/*O*_ data from Table 1. The symbols in black are from the compilation of Savir et al. (2010). The *S*_*C*/*O*_ value for *N. tabacum* L335V mutant is shown. Also on the graph is 〈*S*_*C*/*O*_〉 given by Equation (8) and Equation (10), including a possible factor of Equation (10),$\langle \frac{k_{5}}{k_{11}}\rangle$ (Equation 11).

The graph of reciprocal relative specificity, $S_{O/C}=\frac{1}{S_{C/O}}$, against $k_{cat}^{C}$ (Figure 2D) suggests a linear dependence. The residuals of regressed *S*_*O*/*C*_ data are near normally distributed (Figure 3). We first consider the expected value of *S*_*C*/*O*_ conditional on $k_{cat}^{C}$ as the reciprocal of the equation for the straight line that describes 〈*S*_*O*/*C*_〉, i.e.,

(8) $$\begin{array}{l} \langle S_{C/O}\rangle =\frac{1}{\left(a_{2}+b_{2}k_{cat}^{C}\right)} \end{array}$$

where $a_{2}=7.4\times 10^{-3}\text{ mol}/\text{mol}$ and $b_{2}=1.2\times 10^{-3}s$ are the regression parameters (Figure 2D). Although Equation (8) generally provides a good fit to the data (Figure 6), it clearly does not display the correct limiting behavior as $k_{cat}^{C}$ approaches zero Equation (3). However, defining the expected value as the ratio $\langle S_{C/O}\rangle =\frac{\langle S_{C}\rangle }{\langle S_{O}\rangle }$ and substituting $\langle S_{C}\rangle =\frac{k_{cat}^{C}}{a_{1}e^{b_{1}k_{cat}^{C}}}$ (Figure 2A), the expected value of *S*_*C*/*O*_ conditional on $k_{cat}^{C}$ can be written as

(9) $$\begin{array}{l} \langle S_{C/O}\rangle =\frac{{\langle S_{O}\rangle }^{-1}k_{cat}^{C}}{a_{1}e^{b_{1}k_{cat}^{C}}}. \end{array}$$

**Figure 7:**
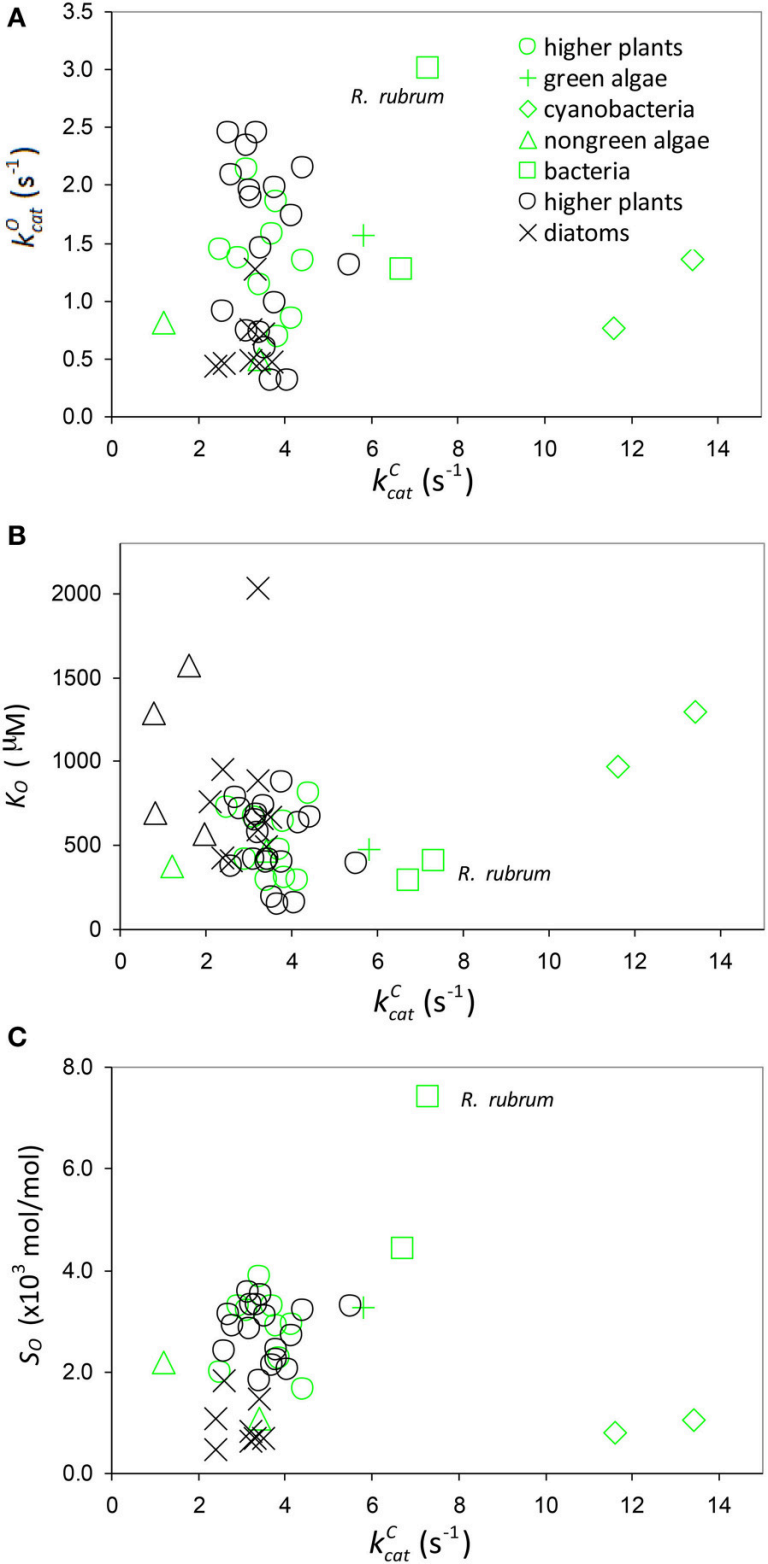
Oxygenation parameters. Scatter plots of **(A)** $k_{cat}^{O}$ against $k_{cat}^{C}$ **(B)** *K*_*O*_ against $k_{cat}^{C}$ and **(C)** specificity, $S_{O}=\frac{k_{cat}^{O}}{K_{O}}$, against $k_{cat}^{C}$ including all data in Table 1. Data points highlighted in green are those compiled by Savir et al. (2010).

As there are no correlations between $k_{cat}^{C}$ and $k_{cat}^{O}$ (Figure 7A) or *K*_*O*_ (Figure 7B), *S*_*O*_ is also not correlated (Figure 7C), and so the best possible approximation for Equation (9) takes the form *S*_*C*/*O*_ ∝ *S*_*C*_. The constant ${\langle S_{O}\rangle }^{-1}$ in Equation (9) can therefore be estimated by a linear regression of *S*_*C*/*O*_ (excluding the outlier, *R. rubrum*, Figure 2D) on $\frac{k_{cat}^{C}}{a_{1}e^{b_{1}k_{cat}^{C}}}$ subject to the constraint *S*_*C*/*O*_ = 0 at $k_{cat}^{C}=0$ to obtain the correct general equation for the expected value of *S*_*C*/*O*_ conditional on $k_{cat}^{C}$ as (Figure 6).

(10) $$\begin{array}{l} \langle S_{C/O}\rangle \approx \frac{490k_{cat}^{C}}{a_{1}e^{b_{1}k_{cat}^{C}}}. \end{array}$$

Assuming correlation (Figure 2B) arises from CO_2_ binding, the factor implicit in Equation (10) corresponding to $\langle \frac{k_{5}}{k_{11}}\rangle$ (Figure 6) that is also conditional on $k_{cat}^{C}$ is estimated by (Equations 4, 7, Figure 4A).

(11) $$\begin{array}{l} \langle \frac{k_{5}}{k_{11}}\rangle \approx 280\langle K_{R}k_{5}\rangle . \end{array}$$

### Mutant example

**Figure 8:**
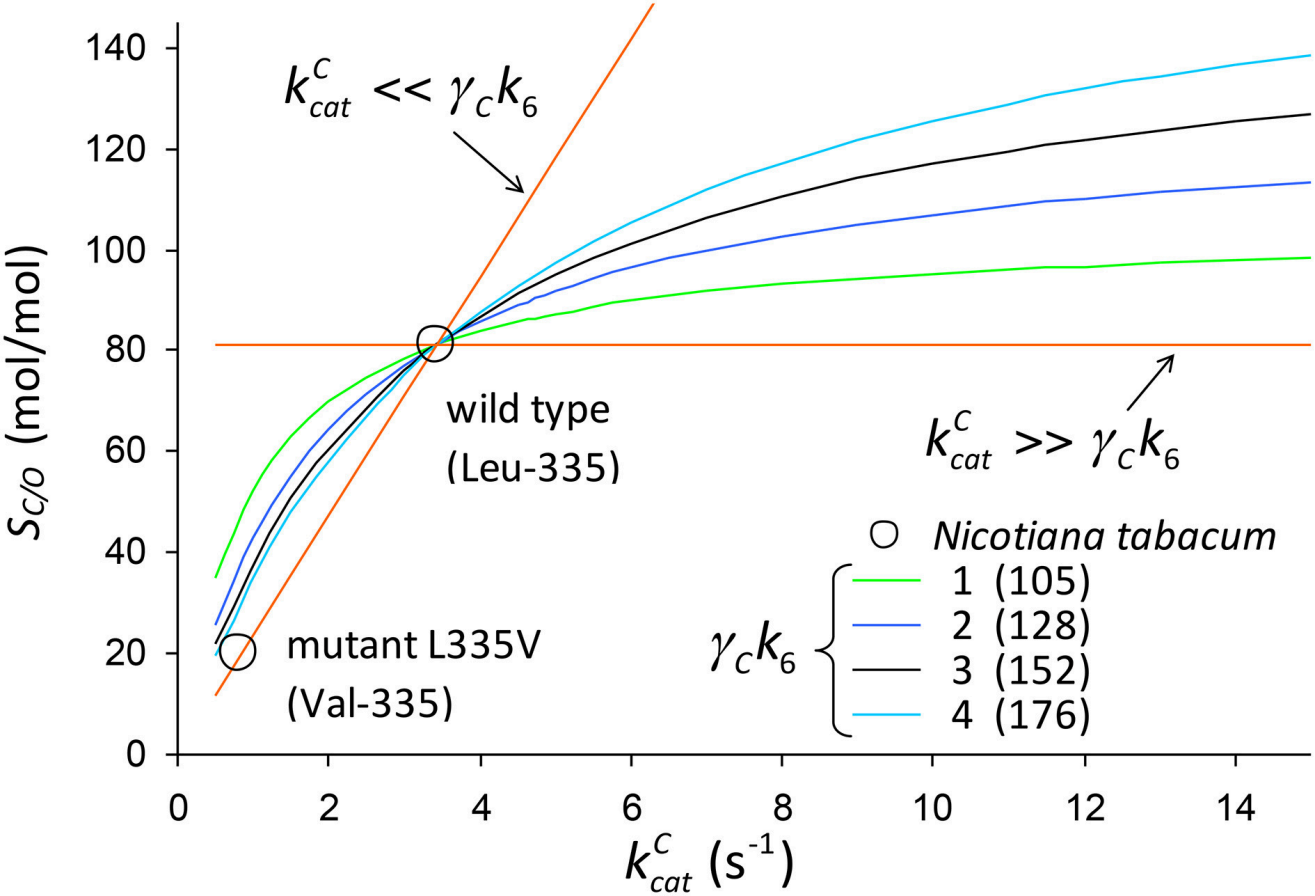
*S*_*C*/*O*_ (Equation 3) plotted against $k_{cat}^{C}$ assuming that $\frac{k_{5}\left(k_{cat}^{O}+\gamma_{O}k_{12}\right)}{k_{11}k_{cat}^{O}}$ is constant on the curve (Equation 12). The numbers in parentheses are the values of $\frac{k_{5}\left(k_{cat}^{O}+\gamma_{O}k_{12}\right)}{k_{11}k_{cat}^{O}}$ in Equation (3) that give *S*_*C*/*O*_ = 81.1 mol/mol for wild-type tobacco given CO_2_ dissociation rate constants of γ_*C*_*k*_6_ = 1, 2, 3 and 4*s*^−1^. For the wild type $k_{cat}^{C}=3.43s^{-1}$, and for the mutant (Val-335) $k_{cat}^{C}=0.81s^{-1}$ and *S*_*C*/*O*_ = 20.1*mol/mol* (Whitney et al., 1999).

We use Equation (3) to rationalize the *in vitro* kinetic data for the Leu to Val mutation at position 335 (L335V) in tobacco (Whitney et al., 1999). The decrease in $k_{cat}^{C}$ from 3.43 s^−1^ in the wild type to 0.81 s^−1^ in the mutant is accompanied by a large decrease also in *S*_*C*/*O*_ from 81 to 20 mol/mol. In Figure 8, *S*_*C*/*O*_ is plotted against $k_{cat}^{C}$ assuming that in Equation (3) the term $\frac{k_{5}\left(k_{cat}^{O}+\gamma_{O}k_{12}\right)}{k_{11}k_{cat}^{O}}$ is constant on the curve, i.e.,

(12) $$\begin{array}{l} S_{C/O}\propto \frac{k_{cat}^{C}}{\left(k_{cat}^{C}+\gamma_{C}k_{6}\right)} \end{array}$$

We determine the constant factor such that *S*_*C*/*O*_ = 81*mol/mol* for the wild-type tobacco at the two limits ($k_{cat}^{C}\gg \gamma_{C}k_{6}$ and $k_{cat}^{C}\ll \gamma_{C}k_{6}$) for specific values of γ_*C*_*k*_6_ = 1, 2, 3 and 4*s*^−1^. Note that in the limit $k_{cat}^{C}\gg \gamma_{C}k_{6}$ we obtain $S_{C/O}=\frac{k_{5}}{k_{11}}=81\text{ mol}/\text{mol}$, while the lower limit for $k_{cat}^{C}\ll \gamma_{C}k_{6}$ gives *S*_*C*/*O*_ = 0. Noting that $\langle \frac{k_{5}}{k_{11}}\rangle =\frac{\langle \frac{K_{R}}{k_{5}}\rangle }{\langle \frac{K_{R}}{k_{11}}\rangle }$, the remaining kinetic parameters [*K*_*C*_ = 10.7 μM, $k_{cat}^{O}=1.17s^{-1}$, *K*_*O*_ = 295 μM for wild type, and *K*_*C*_ = 5.1 μM, $k_{cat}^{O}=0.39s^{-1}$, *K*_*O*_ = 48.9 μM for the mutant] (Whitney et al., 1999) can be used to simply determine the expected value of the ratio $\frac{k_{5}}{k_{11}}$ as.

(13) $$\begin{array}{l} \langle \frac{k_{5}}{k_{11}}\rangle =\frac{\Delta k_{cat}^{C}K_{O}}{\Delta k_{cat}^{O}\Delta K_{C}}\approx 150 \end{array}$$

where Δ is the difference between wild type and mutant.

## Discussion

### Significant dissociation of CO_2_ and O_2_ substrates

The trend lines (Figure 2) clearly intercept the vertical axes well above zero, indicating significant expected values for the dissociation constants γ_*C*_*k*_6_ and γ_*O*_*k*_12_. However, the rate constant for CO_2_ dissociation has been previously estimated as not more than about 5% of $k_{cat}^{C}$ (Pierce et al., 1986; McNevin et al., 2007), so that it has generally been assumed that $\frac{k_{cat}^{C}}{\left(k_{cat}^{C}+\gamma_{C}k_{6}\right)}\approx 1$. Our estimates (Figures 2A,B, Table 3) of the expected value (at least for low $k_{cat}^{C}$) are much higher, and find support in the kinetics modeling study of RuBisCO from spinach. We find that the expected values of dissociation rate constants (γ_*C*_*k*_6_) for the binding of the substrate CO_2_ are 4.3 s^−1^ (Figure 2A), 3.0 s^−1^ (Figure 2B), and 3.1 s^−1^ for a subset of C_3_ plants (Galmés et al., 2014; Prins et al., 2016), noting that the differences are not statistically significant (Table 3). These values can be compared with 1.6 ±1.1 s^−1^ estimated for the CO_2_ dissociation rate constant in spinach (McNevin et al., 2006, 2007), and the 5−10 μM range of $\langle K_{D}^{C}\rangle$ for lower values of $k_{cat}^{C}$ (Figures 4B, 5B) is also consistent with a $K_{D}^{C}=\frac{k_{6}}{k_{5}}$ of 3 μM for spinach RuBisCO (McNevin et al., 2006). The effective CO_2_ dissociation rate constant, γ_*C*_*k*_6_, impacts the $k_{cat}^{C}$ dependence of *S*_*C*/*O*_ (Figure 8). As $k_{cat}^{C}$ approaches γ_*C*_*k*_6_ Equation (12) describes the rapid decline in *S*_*C*/*O*_ due to increasing probability that the CO_2_ will dissociate from RuBP before catalysis takes place. The observed values of $k_{cat}^{C}$ and *S*_*C*/*O*_ for the L335V mutant (Whitney et al., 1999) are entirely consistent with a γ_*C*_*k*_6_ greater than $k_{cat}^{C}$. The expected value of $\frac{k_{5}}{k_{11}}$ as given by Equation (13) is also consistent with the value obtained when averaged over a larger number of RuBisCOs with lower $k_{cat}^{C}$ (Figure 6). Thus, changes in the gas-substrate binding in the mutant RuBisCO appear to be minimal, the bulk of the effect being described by Equation (12). The dissociation rate constant of O_2_ is generally considered effectively zero (Tcherkez, 2013, 2016). However, although the expected value of 0.4 ± 0.4 s^−1^ for γ_*O*_*k*_12_ in higher plants obtained here (Table 3) is significantly lower than the mean $k_{cat}^{O}$ of 1.3 ± 0.2 s^−1^ (from data in Table 1) it is still sufficient to have an impact on *K*_*O*_ (Equation 2). Additionally, the expected value of 2.3 ± 1.9 s^−1^ for γ_*O*_*k*_12_ in Triticeae (Table 3) and the corresponding mean $k_{cat}^{O}$ of 0.83 ± 0.16 s^−1^ (Prins et al., 2016) are not significantly different. Statistical analysis of the available data therefore suggests the expected (or average) value of the dissociation rate is not significantly lower than that of the catalytic rate. Moreover, a knowledge of rate differences in any particular RuBisCO requires more kinetic data than is currently available. Consequently, there is no justification for generally neglecting either of the dissociation rate constants, γ_*C*_*k*_6_ or γ_*O*_*k*_12_, i.e., assuming they are an order of magnitude or more lower than the corresponding catalytic rates, as has been done previously (Tcherkez, 2013, 2016).

### The tight-binding hypothesis

Assuming $\langle \frac{k_{5}}{k_{11}}\rangle$ decreases with increasing $k_{cat}^{C}$ (Equation 11, Figure 6), it could be regarded as a proxy for *S*_*C*/*O*_ (Tcherkez, 2013). Also as the specificity of oxygenation, *S*_*O*_, is not correlated with $k_{cat}^{C}$ (Figure 7C), the variation in $\langle \frac{k_{5}}{k_{11}}\rangle$ would be largely constrained to the dependence of 〈*k*_5_〉 on $k_{cat}^{C}$ (Figure 4A). It has been hypothesized (Tcherkez et al., 2006; Tcherkez, 2013) that such a constraint is to be expected from the predicted energetics of the reaction as tighter binding of CO_2_ to ribulose bisphosphate (increasing *k*_5_) would necessarily raise the activation free energy (decreasing $k_{cat}^{C}$) required for the subsequent steps leading to turnover of product. However, the generality of this tight-binding (TB) hypothesis has come under question (Hanson, 2016) for its inability to explain the variations in *S*_*C*/*O*_ that have been observed in some RuBisCOs (Young et al., 2016). It would seem that the TB hypothesis suffers from a more fundamental problem in that it is based on an incomplete and unrepresentative data distribution. In the present analysis, Equation (8) provides the better fit *R*^2^ = 0.63 to the selected data (Figure 2D), although it is not the more general equation for 〈*S*_*C*/*O*_〉 (Equation 10, Figure 6). Similar types of relationships that provide an even tighter fit to the data have been reported elsewhere: $K_{C}\propto {\left(k_{cat}^{C}\right)}^{2}$ [*R*^2^ = 0.90] and $S_{C/O}\propto {\left(k_{cat}^{C}\right)}^{-0.51}$ [*R*^2^ = 0.79] (Savir et al., 2010). The TB hypothesis is posited on $\frac{k_{5}}{k_{11}}$ determining the dependence of *S*_*C*/*O*_ on $k_{cat}^{C}$. Significantly, all of these analyses are in fact conditional on $k_{cat}^{C}\gg \gamma_{C}k_{6}$, i.e, neglect of the CO_2_ dissociation rate constant, *k*_6_. However, the high level of variance in *K*_*C*_ and *S*_*C*/*O*_ (Figures 2A, 6, respectively) argues for a more cautious data interpretation in the regression analysis. Statistically, the quadratic (Savir et al., 2010) and exponential (Figure 2B) forms both describe the dependence of *K*_*C*_ on $k_{cat}^{C}$ equally well, but only the latter, more general case (Equations 3, 10), allows nonzero values for γ_*C*_*k*_6_ (Figure 5A).

### Rate constants may not be highly correlated

The deviation of any given data point (Figure 6) from the expected value (Equation 10) can be attributed to variations in the parameters of Equation (3). We expect that *S*_*O*_ will generally produce random variations in *S*_*C*/*O*_ (Figure 7C), although, possibly lower *k*_11_ (higher *K*_*O*_, Figure 2C) for the cyanobacteria may in part account for a systematic reduction in *S*_*C*/*O*_. The CO_2_ dissociation term, γ_*C*_*k*_6_, will certainly become apparent at low enough $k_{cat}^{C}$ values (Figures 4, 5). In particular, variations in γ_*C*_*k*_6_ may contribute significantly to the large variance seen in the non-green algae (Figures 2A, 6). If the catalytic rate correlates with *k*_5_, regression analysis defines only the first moment, 〈*k*_5_〉, of the distribution (Figure 4A and Equation 11, Figure 6), and provides no information on the variance. In the absence of any coupling, mutations produce random changes in the underlying rate constants, *k*_*i*_. Irrespective of whether rate constants are correlated, the expected value of *k*_*i*_ is given by $\frac{\overset{n}{\underset{s}{\sum }}k_{i}^{s}}{n}$ where $k_{i}^{s}$ is the value of a rate constant for a given sequence (*s*). In reality, the composition of the sequence space, Ω (i.e., any number of known sequences), will be determined in varying degrees by genetic drift and natural selection, as these determine the probability that a mutation becomes fixed. If the variations in $k_{i}^{s}$ themselves are entirely random (zero correlation), we might expect both *S*_*C*/*O*_ and $k_{cat}^{C}$ at the high end of their observed values, as there is nothing to constrain them and the combined effect should have become fixed in some species by positive selection. The TB hypothesis attempts to explain this absence of both high *S*_*C*/*O*_ and high $k_{cat}^{C}$ by positive selection processes occurring within particular constraints (Figures 4A, 6) imposed on the chemical reaction steps (Tcherkez et al., 2006), but it may also be explained by competing selection pressures. The essential difference is that the origin of the evolutionary constraints is shifted from $k_{i}^{s}$ to Ω.

### Competing selection pressures may constrain RuBisCo

From a biophysical perspective, thermodynamic stability is recognized as the most important constraint on the evolution of proteins and their ability to acquire new function (Tokuriki and Tawfik, 2009; Sikosek and Chan, 2014). The necessity of a protein to maintain the integrity of its folded structure despite the destabilizing effects of accumulated mutations results in only a small percentage being fixed by positive selection. Consequently, in the evolution of C_3_ to C_4_ plants, destabilizing mutations that are selected on the basis of improved activity are followed by mutations that restore stability with little impact on activity (Studer et al., 2014). This leads to an apparent tradeoff between activity and stability that may well limit the ability of RuBisCO to fix the number of mutations required to increase both *S*_*C*/*O*_ and $k_{cat}^{C}$. Depending on the sub-cellular CO_2_/O_2_ ratio, the fixed mutations increase specificity (for low ratio) or catalytic rate (for high ratio), or a varying combination of both, whichever best optimizes photosynthesis.

### Potential for optimizing carbon fixation

The origin of the constraint(s) has significant implications for the optimization of RuBisCO activity. If the constraint is on Ω (i.e., from competing selection pressures) rather than $k_{i}^{s}$, greater variability may be exhibited. To what extent the functional limits of RuBisCO are reflected in the minimum and maximum values of kinetic parameters is not yet clear for RuBisCOs with higher $k_{cat}^{C}$ because of the absence of empirical data. Much effort has been directed toward research on higher plants with particular emphasis on the evolution of C_3_ to C_4_ plants with their associated CCMs, although the recent work on diatoms may now help stimulate investigations into a more diverse range of photosynthetic organisms (Hanson, 2016; Young et al., 2016). Diatoms and C_3_ plants share very similar $k_{cat}^{C}$, although the variance, var(*S*_*C*/*O*_), for diatoms is relatively large (with corresponding variations in CCM expression), whereas for C_3_ plants var(*S*_*C*/*O*_) is barely significant (Figure 6, Table 1). This could raise the possibility of improving specificity, if not $k_{cat}^{C}$, in higher plants. It is perhaps not surprising that the non-green (red) algae, from which diatoms have evolved with somewhat lower $k_{cat}^{C}$ values, also exhibit high var(*S*_*C*/*O*_) (Figure 6). The data distributions are incomplete (Figures 2A, 6, Table 1); there is a scarcity of data for green algae, photosynthetic bacteria and cyanobacteria, with $k_{cat}^{C}$ values between 6 s^−1^ and 14 s^−1^. Discoveries of significant variance among these also may provide important clues on how to achieve increases in both $k_{cat}^{C}$ and *S*_*C*/*O*_ in higher plants.

## Conclusion

The results of our analysis using regression analysis on updated RuBisCO-kinetic data sets suggest that CO_2_ dissociation from the RuBisCO gas-addition complex is generally more important in rationalizing the observed variations in the kinetics of RuBisCO than hitherto assumed (Tcherkez et al., 2006; Tcherkez, 2013). Moreover, we have identified significant variations in the statistical correlations between *K*_*M*_ and *k*_*cat*_ in higher plants, i.e., the non-linear correlation for carboxylation as opposed to the linear correlation for oxygenation. These findings cast doubt on the hypothesis (Tcherkez et al., 2006; Savir et al., 2010; Tcherkez, 2013) that RuBisCO is so tightly constrained by the active-site chemistry that its activity is effectively optimized. Rather, the current body of kinetic parameters exhibits far more plasticity than this hypothesis predicts. We suggest that the possibility that the apparent tradeoff observed between $k_{cat}^{C}$ and *S*_*C*/*O*_ could arise from competing selection pressures on RuBisCO activity and stability (Studer et al., 2014) be given more attention. The relative strengths of these selection pressures would determine the strength of the constraints and, thus, the possibilities of improving the kinetics of RuBisCO by site-directed mutagenesis. Indeed, although published comments (Griffiths, 2006; Gutteridge and Pierce, 2006) on the paper of Tcherkez et al. (2006) noted the vastness of sequence space that would need to be sampled, neither showed any positivity that a rational method to increase the efficiency of such a search was possible merely noting (Griffiths, 2006) directed evolution as a possibility. However, a method to reduce the sequence-search space for RuBisCO has since been reported in a patent (Gready and Kannappan, 2009).

In summary, there is still wide conjecture in the literature regarding the mechanisms by which plants ultimately regulate photosynthesis (Igamberdiev, 2015), and the absolute limitations of RuBisCO functionality have only been partly explored, as recent studies (Hanson, 2016; Young et al., 2016) suggest. Consequently, the potential for increasing both the catalytic turnover and relative specificity in higher plants with the view to improving photosynthesis remains to be fully tested. As argued (Hanson, 2016), kinetic data for a wider diversity of RuBisCOs are much needed and will likely prove useful in guiding the reengineering of higher-plant RuBisCOs with both significantly higher turnover rate and specificity. Our analysis suggests that such simultaneous improvement in both specificity and turnover rate is possible, and that competing selection pressures of activity and stability better explain the nature of constraints. Improved understanding of these competing selection pressures is much needed.

## Author contributions

PC, BK, and JG designed and performed the research, wrote the paper and approved it for submission.

### Conflict of interest statement

The authors declare that the research was conducted in the absence of any commercial or financial relationships that could be construed as a potential conflict of interest.
