# Supplementary material for: Directions for Optimization of Photosynthetic Carbon Fixation: RuBisCO's Efficiency May Not Be So Constrained After All
Source: Front Plant Sci. 2018 Mar 1;9:183. doi: 10.3389/fpls.2018.00183 (PMC5838012; doi:10.3389/fpls.2018.00183)
Supplement: Supplementary file 1 [file DataSheet1.docx]

**Supplementary Material**

**for**

**Directions for optimization of photosynthetic carbon fixation: RuBisCO’s efficiency may not be so constrained after all**

Peter L. Cummins, Babu Kannappan, and Jill E. Gready^*^

John Curtin School of Medical Research, The Australian National University,

Canberra ACT Australia

^*^ To whom correspondence should be addressed. E-mail: jill.gready@anu.edu.au

**Appendix**

**Derivation of the kinetic equations**: The concentrations used in the kinetic equations are *E*: activated form of the enzyme; *R*: unbound RuBP; *ER*: RuBisCO…RuBP complex; *ER**: RuBisCO…enediolate of RuBP complex; *C*: free CO_2_; *O*: free O_2_; *ERC*: RuBisCO…carboxylated intermediate complex; *ERO*: RuBisCO…oxygenated intermediate complex; *ERP*: RuBisCO…carboxylated product complex; *ERX*: RuBisCO…oxygenated product complex; *G*: 3-phosphoglyceric acid; *Q*: 2-phospho-glycolate.

The mass balance equation for the kinetic mechanism (Fig. 1) is given by (*E_t_* is the total activated enzyme concentration)

*E* + *ER* + *ER** + *ERC* + *ERO* + *EP* + *EX* – *E_t_* = 0 (A1)

The steady state ordinary differential equations (ODEs) for this kinetic scheme are

$${d ER}/{dt=k_{1}}E\cdot R-\left( k_{2}+k_{3} \right)ER+k_{4}{ER}^{*}=0$$

(A2)

$${d {ER}^{*}}/{dt=k_{3}}ER-\left( k_{4}+k_{5}C+k_{11}O \right){ER}^{*}+k_{6}ERC+k_{12}ERO=0$$

(A3)

${d ERC}/{dt=k_{5}}{C\cdot ER}^{*}-\left( k_{6}+k_{7} \right)ERC+k_{8}EP=0$ (A4)

${d EP}/{dt=k_{7}}ERC-\left( k_{8}+k_{9} \right)EP+k_{10}E\cdot G\cdot G=0$ (A5)

${d ERO}/{dt=k_{11}}{O\cdot ER}^{*}-\left( k_{12}+k_{13} \right)ERO+k_{14}EX=0$ (A6)

${d EX}/{dt=k_{13}}ERO-\left( k_{14}+k_{15} \right)EX+k_{16}E\cdot G\cdot Q=0$ (A7)

It is convenient to define the following constants:

$\alpha_{C}=\left( k_{9}+k_{8} \right)/{k_{7}}$

$\alpha_{O}=\left( k_{15}+k_{14} \right)/{k_{13}}$

$\beta_{C}=k_{9}+k_{6}\alpha_{C}$

$\beta_{O}=k_{15}+k_{12}\alpha_{O}$

From the above steady state ODEs we can readily express the concentrations of free enzyme *E* and all reaction intermediates in terms of the product complexes (either *EP* or *EX*). For the carboxylation reaction (*EP*) we obtain (assuming only that product release is “irreversible” i.e.):

Summing (A2) to (A7): $E=\frac{k_{2}ER+k_{9}EP+k_{15}EX}{k_{1}R}$ (A8)

From (A5): *ERC* = *EP* (A9)

From (A7): *ERO* = *EX* (A10)

From (A4) + (A5) and (A9): $ER*=\frac{k_{9}EP+k_{6}ERC}{k_{5}C}=\frac{\beta_{C}}{k_{5}C}EP$ (A11)

From (A6) + (A7), (A10) and (A11): $EX=\frac{k_{11}O\cdot ER*}{\beta_{O}}=\frac{\beta_{C}k_{11}O}{\beta_{O}k_{5}C}EP$ (A12)

From (A10) and (A12):$ERO=\alpha_{O}EX=\frac{\alpha_{O}\beta_{C}k_{11}O}{\beta_{O}k_{5}C}EP$ (A13)

From (A2), (A8), (A11) and (A12):$ER=\frac{k_{9}EP+k_{15}EX+k_{4}ER*}{k_{3}}$

$=\frac{k_{9}}{k_{3}}EP+\frac{\beta_{C}k_{11}k_{15}O}{\beta_{O}k_{3}k_{5}C}EP+\frac{k_{4}\beta_{C}}{k_{3}k_{5}C}EP$ (A14)

Substituting (A8)-(A14) into (A1) and factorizing we get the steady state equation in the form

$\frac{E_{t}}{EP}=1+f_{1}^{C}+\frac{f_{2}^{C}}{R}+\frac{f_{3}^{C}}{C}+\frac{f_{4}^{C}}{RC}$ (A15)

where the coefficients$f_{i}^{C}$are given by

$f_{1}^{C}=\frac{k_{9}+k_{3}\alpha_{C}}{k_{3}}$ (A16a)

$f_{2}^{C}=\frac{k_{9}(k_{2}+k_{3})}{k_{1}k_{3}}$ (A16b)

$f_{3}^{C}=\frac{\beta_{C}}{k_{3}k_{5}}\left(k_{3}+k_{4}+\frac{(k_{3}+k_{15}+k_{3}\alpha_{O})k_{11}O}{\beta_{O}}\right)$ (A16c)

$f_{4}^{C}=\frac{\beta_{C}}{k_{1}k_{3}k_{5}}\left(k_{2}k_{4}+\frac{(k_{2}+k_{3})k_{11}k_{15}O}{\beta_{O}}\right)$ (A16d)

The rate of CO_2_ consumption is given by

$V_{C}=-\frac{dC}{dt}=k_{5}C\cdot ER*-k_{6}ERC=(k_{9}+k_{6}\alpha_{C})EP-k_{6}\alpha_{C}EP=k_{9}EP$ (A17)

Rewriting (A15) in terms of$EP$ and substituting the result into (A17) gives

$V_{C}=\frac{k_{9}E_{t}RC}{f_{4}^{C}+f_{3}^{C}R+f_{2}^{C}C+(f_{1}^{C}+1)RC}$ (A18)

When both substrates, *R* and *C*, are saturating the maximum rate of CO_2_ consumption,$V_{\max}^{C}$, is obtained as,

$V_{\max}^{C}=\frac{k_{9}}{1+f_{1}^{C}}E_{t}=k_{cat}^{C}E_{t}$ (A19)

Substituting (A16a) into (A19) and rearranging we get for $k_{cat}^{C}$ in terms of the rate constants$k_{i}$

$$k_{cat}^{C}=\frac{k_{3}k_{7}k_{9}}{k_{3}k_{7}+k_{3}k_{8}+k_{3}k_{9}+k_{7}k_{9}}$$

Finally, rewriting (A18) in terms of $V_{\max}^{C}$gives the familiar (e.g. ([Farquhar, 1979](#_ENREF_10))) general form of the steady state rate equation,

$V_{C}=\frac{V_{\max}^{C}R\cdot C}{K_{3}^{C}+K_{2}^{C}R+K_{1}^{C}C+R\cdot C}$ (A20)

where $K_{i}^{C}=f_{i+1}^{C}/(1+f_{1}^{C})$. (A21)

It immediately follows that the rate of oxygen consumption by the enzyme can be written as

$$V_{O}=\frac{V_{\max}^{O}R\cdot O}{K_{3}^{O}+K_{2}^{O}R+K_{1}^{O}O+R\cdot O}$$

where $V_{\max}^{O}=k_{cat}^{O}E_{t}$ and $k_{cat}^{O}=\frac{k_{15}}{1+f_{1}^{O}}=\frac{k_{3}k_{13}k_{15}}{k_{3}k_{13}+k_{3}k_{14}+k_{3}k_{15}+k_{13}k_{15}}$.

The Michaelis-Menten equation (A20) for the single substrate *C* when *R* is saturating becomes

$$V_{C}=\frac{V_{\max}^{C}C}{K_{2}^{C}+C}$$

From (A21), (A16a) and (A16c):

$K_{2}^{C}=\frac{f_{3}^{C}}{1+f_{1}^{C}}=\frac{\beta_{C}}{(1+f_{1}^{C})k_{3}k_{5}}\left[k_{3}+k_{4}+\frac{(k_{3}+k_{15}+k_{3}\alpha_{O})k_{11}O}{\beta_{O}}\right]$ (A22)

Substituting$f_{1}^{O}=\frac{k_{15}+k_{3}\alpha_{O}}{k_{3}}$, $K_{R}=\frac{k_{3}}{k_{3}+k_{4}}$, $\gamma_{C}=\frac{\alpha_{C}}{1+f_{1}^{C}}$ and $\gamma_{O}=\frac{\alpha_{O}}{1+f_{1}^{O}}$

into (A22) yields the Michaelis constant in the presence of the other (*O*) substrate

$$K_{2}^{C}=K_{C}\left(1+\frac{O}{K_{O}}\right)$$

where $K_{C}=\frac{k_{9}+k_{6}\alpha_{C}}{(1+f_{1}^{C})K_{R}k_{5}}=\frac{k_{cat}^{C}+\gamma_{C}k_{6}}{K_{R}k_{5}}$ (A23)

and $K_{O}=\frac{k_{15}+k_{12}\alpha_{O}}{(1+f_{1}^{O})K_{R}k_{11}}=\frac{k_{cat}^{O}+\gamma_{O}k_{12}}{K_{R}k_{11}}$ (A24)

The specificities of each of the reactions are then

$S_{C}=\frac{k_{cat}^{C}}{K_{C}}=\frac{k_{cat}^{C}K_{R}k_{5}}{k_{cat}^{C}+\gamma_{C}k_{6}}$ and $S_{O}=\frac{k_{cat}^{O}}{K_{O}}=\frac{k_{cat}^{O}K_{R}k_{11}}{k_{cat}^{O}+\gamma_{O}k_{12}}$

and so the specificity of carboxylation relative to oxygenation (relative specificity) is given by

$S_{C/O}=\frac{S_{C}}{S_{O}}=\frac{k_{5}k_{cat}^{C}(k_{cat}^{O}+\gamma_{O}k_{12})}{k_{11}k_{cat}^{O}(k_{cat}^{C}+\gamma_{C}k_{6})}$ (A25)

In terms of the rate constants we find the coefficients of $k_{6}$ and $k_{12}$:

$\gamma_{C}=\frac{\alpha_{C}}{1+f_{1}^{C}}=\frac{(k_{8}+k_{9})k_{cat}^{C}}{k_{7}k_{9}}=\frac{k_{3}k_{8}+k_{3}k_{9}}{k_{3}k_{7}+k_{3}k_{8}+k_{3}k_{9}+k_{7}k_{9}}$ (A26)

$\gamma_{O}=\frac{\alpha_{O}}{1+f_{1}^{O}}=\frac{(k_{14}+k_{15})k_{cat}^{O}}{k_{13}k_{15}}=\frac{k_{3}k_{14}+k_{3}k_{15}}{k_{3}k_{13}+k_{3}k_{14}+k_{3}k_{15}+k_{13}k_{15}}$ (A27)

Thus the range of $\gamma$ is limited to [0,1].
